# Supplementary material for: The Relationship between Fluoride Exposure and Cognitive Outcomes from Gestation to Adulthood—A Systematic Review
Source: Int J Environ Res Public Health. 2022 Dec 20;20(1):22. doi: 10.3390/ijerph20010022 (PMC9819484; doi:10.3390/ijerph20010022)
Supplement: Supplementary file 1 [file ijerph-20-00022-s001.zip › ijerph-2055424-supplementary/Supplementary File S1.pdf]

Supplementary File S1 - MEDLINE Search Strategy

#	**Query**
S1	(Child* OR Infant* OR Pregnan* OR Preschool* OR “Pre school*” OR Antenatal OR Mother* OR Pediatric OR Paediatric OR Paediatric OR Baby OR Toddler OR Newborn OR Fetus OR Foetus OR Embryo OR adolescen* OR juvenil* OR youth* OR teen* OR preteen* OR “pre teen*” OR pubescen* OR schoolchild* OR kid* OR boy* OR girl* OR early life OR young adult*)
S2	(Fluori*)
S3	(Dentifrice OR Toothpaste OR “toothpaste*” OR Mouthwash OR “mouth wash”)
S4	S2 OR S3
S5	(Cognitive develop* OR Cognitive impair* OR IQ OR Neurotoxic* OR neurologic OR Cognition OR Intelligen* OR ADHD OR Hyperactiv* OR Attention deficit disorder OR Downs syndrome OR Learning disab* OR Learning Disorder* OR developmental disab* OR intellectual disabl* OR mental* retard* OR Impaired brain function OR Dyslexia OR Brain growth OR Brain development OR Cognitive control OR Working memory OR Inhibitory control OR Reasoning OR Problem solving OR Neurocognit* OR Self-regulation OR Selfregulation OR School readiness OR Kindergarten readiness OR Intelligence quotient OR "Educational Measurement" OR "educational outcome" OR academic* OR "achievement gap*" OR "student performance" OR "time on task" OR "standardized test*" OR "reading achievement" OR "reading performance" OR "reading assessment*" OR "math* achievement" OR "math* performance" OR "math* assessment*" OR "science performance" OR "science achievement" OR "science assessment*" OR "writing performance" OR "writing achievement" OR "writing assessment" OR "test* score*" OR exam* OR "exam score*" OR "classroom learning" OR "classroom performance" OR “attention problem”)
S6	S1 AND S4 AND S5
