# Supplementary material for: The Relationship between Fluoride Exposure and Cognitive Outcomes from Gestation to Adulthood—A Systematic Review
Source: Int J Environ Res Public Health. 2022 Dec 20;20(1):22. doi: 10.3390/ijerph20010022 (PMC9819484; doi:10.3390/ijerph20010022)
Supplement: Supplementary file 1 [file ijerph-20-00022-s001.zip › ijerph-2055424-supplementary/Supplementary File S2.pdf]

Table S2. Summary of data extraction from included studies.

Study Reference	Year, Country	Study design and setting	Initial sample size, age (year) and % male	Cognitive assessment tool	Methods of assessment of Fluoride exposure	Water fluoride level at baseline (mg/L)	Results (Outcome and significance)	Study Quality (score and classification)
**Aravind et al. [45]**	2016 India	Cross -sectional<br><br>School	288<br><br>10 - 12<br><br>49%	Raven's Standard Progressive Matrices	Drinking water	<1.2<br><br>1.2 – 2<br><br>> 2	Mean IQ (41.03±16.36)<br><br>(56.68±14.51)<br><br>(31.59±16.81)<br><br>Significance: $p<0.0001$<br>The mean IQ for medium F was significantly greater than low/high F<br><br>Negative Association	63.6<br><br>Fair
**Barberio et al. [60]**	2017 Canada	Cross -sectional<br><br>Community	2221<br><br>6.9<br><br>50.5%	Canadian Health Measures Survey Questionnaire	Drinking water, Maternal urinary fluoride, Fluoride containing product, Fluoride treatment at dentist	0.23	Self-reporting learning disability<br><br>7.58 (95% CI: 4.80–10.37)<br><br>3.78 (95% CI: 1.34–6.23)<br><br>Significance: Positive Association	85.7<br><br>Excellent
**Bashash et al. [55]**	2017 Mexico	Longitudinal<br><br>Community	785<br><br>6.27<br><br>44.5%	McCarthy Scales of Children's Abilities. Spanish version of the Wechsler Abbreviated Scale of Intelligence. Wechsler Adult Intelligence Scale – Spanish	Drinking water, Maternal Urinary fluoride	0.15 to 1.38	Mean GCI = 96.88, Mean IQ =95.98<br><br>Significance: $p < 0.01$ Positive Association	85.7<br><br>Excellent

Study Reference	Year, Country	Study design and setting	Initial sample size, age (year) and % male	Cognitive assessment tool	Methods of assessment of Fluoride exposure	Water fluoride level at baseline (mg/L)	Results (Outcome and significance)	Study Quality (score and classification)
**Bashash et al. [54]**	2018<br>Mexico	Longitudinal<br><br>Community	420<br><br>8.4<br><br>46%	Spanish version of the Conners Rating Scale – Revised. Conners' Continuous Performance Test (CPT-II, 2nd Edition)	Drinking water,<br>Maternal urinary fluoride,<br>Salt F (250 ± 50 µg/g)	0.15 to 1.38	Mean IQ = 54.62 [p 0.88]<br><br>Mean IQ = 55.61 [p 0.67]<br><br>Significance: Negative association between MuF and CRS-R scales.<br><br>Positive association between MuF and CPT-II	81.8<br><br>Good
**Broadbent et al. [66]**	2015<br>New Zealand	Longitudinal<br><br>Community	1032<br><br>7 - 13<br><br>52%	Wechsler Adult Intelligence Scale – Revised. Wechsler Adult Intelligence scale - Fourth Edition	Community water fluoridation area,<br>Fluoride tablets (0.5-mg),<br>Fluoridated toothpaste	Residents with CWF 0.7-1.0<br><br>Residents without CWF 0.0 - 0.3	Mean IQ at age 7–13 = 100.0<br><br>IQ at age 38 years = 100.2<br><br>Significance: Positive Association<br>p < 0.001	71.4<br><br>Good
**Chen et al. [22]**	2008<br>China	Cross - sectional<br><br>Community	640<br><br>10.5<br><br>50%	Chinese Standardized Raven Test	Drinking water	4.55	Mean IQ = 100.24±14.52<br><br>Significance: p < 0.01<br>Negative Association	32.5<br><br>Poor
**Choi et al. [23]**	2015<br>China	Cross -sectional<br><br>School	51<br><br>7.1<br><br>47%	The Wide Range Assessment of Memory and Learning (WRAML),<br>The Wechsler Intelligence Scale for	Drinking water,<br>Urinary Fluoride	1.0–4.07	Total Wide Range Assessment of Visual Motor Ability<br>10.7 (2.7)	63.6<br><br>Fair

Study Reference	Year, Country	Study design and setting	Initial sample size, age (year) and % male	Cognitive assessment tool	Methods of assessment of Fluoride exposure	Water fluoride level at baseline (mg/L)	Results (Outcome and significance)	Study Quality (score and classification)
				Children-Revised (WISC-IV)			Significance: p < 0.001<br>Negative Association	
**Chunyuan et al. [24]**	2011<br>China	Longitudinal<br><br>Community	1400<br><br>7.5<br><br>NR	Strengths and Difficulties Questionnaire (SDQ) and the Developmental Coordination Disorder Questionnaire (DCDQ)	Drinking water, Maternal Plasma,	NR	Mean DCDQ - 67.8 ± 8.1<br><br>Significance: p < 0.01<br>Positive Association	64.9<br><br>Fair
**Cui et al. [25]**	2018<br>China	Cross - sectional<br><br>School	323<br><br>7 - 12<br><br>54.8%	Combined Raven's Test- The rural in China method	Drinking water, Urinary Fluoride	1.52-2.49	IQ scores ≤ 9 years old = 117.4 (12.3),<br><br>IQ scores > 9 years old = 110.0 (12.2)]<br><br>Significance: p < 0.02<br>Negative Association	85.7<br><br>Excellent
**Ding et al. [26]**	2011<br>China	Cross - sectional<br><br>School	331<br><br>7 - 14<br><br>50.1%	Combined Raven's Test-<br>The Rural in China method	Drinking water, Urinary Fluoride	1.31±1.05	IQ score (Mean ± SD), Normal = 103.77 ± 13.61,<br>Very mild = 101.70 ± 13.68,<br>Mild = 107.36 ± 13.62,<br>Moderate = 103.27 ± 13.68<br><br>Significance: p < 0.0001<br>Negative Association	58.4<br><br>Fair

Study Reference	Year, Country	Study design and setting	Initial sample size, age (year) and % male	Cognitive assessment tool	Methods of assessment of Fluoride exposure	Water fluoride level at baseline (mg/L)	Results (Outcome and significance)	Study Quality (score and classification)
**Eswar et al. [46]**	2011 India	Cross – sectional<br>School	133<br>12 – 17<br>52.9%	Standard Progressive Matrices test	Drinking water	0.29<br>2.45	Mean IQ = 88.8±15.3, Mean IQ = 86.3±12.8<br>Significance: p < 0.3 Positive Association	44.2<br>Poor
**Green et al. [61]**	2019 Canada	Longitudinal<br>Community	162<br>3.49<br>49%	Wechsler Preschool and Primary Scale of Intelligence, Third Edition. Full scale IQ(FSIQ), Verbal IQ, Performance IQ	Drinking water, Maternal urinary fluoride	0.59	FSIQ = 108.21<br>Significance: p < 0.001 Negative Association	89.6<br>Excellent
**Guo et al. [27]**	2008 China	Cross – sectional<br>School	121<br>7 - 13<br>50%	Chinese Binet IQ Test	Drinking water, Coal burning (118.11 - 1361.70 mg/kg in coal); 0.0298 mg/m3 in air indoors)	0.5	Mean IQ = 76.7; 81.4<br>Significance: p < 0.05 Negative Association	31.2<br>Poor
**Jimenez et al. [56]**	2017 Mexico	Longitudinal<br>Community	65<br>22 and 43 weeks<br>30.7%	Bayley Scale of Infant Development II (BSDI-II)	Drinking water	Tap water = 0.5 to 12.5; Bottled water = 0.01 – 8.1	Mental Development Index (MDI) – 91.6±14.3 (60.0 - 135.0); Psychomotor Development Index (PDI) - 90.9±13.5 (54.0 - 131.0)<br>Significance: p < 0.013 Negative Association	74.0<br>Good
**Karimzade et al. [63]**	2014 Iran	Cross-sectional<br>Community	39<br>10.68	Raymond B Cattell test	Drinking water	3.94	IQ Level = 81.21±16.17,	41.6<br>Poor

Study Reference	Year, Country	Study design and setting	Initial sample size, age (year) and % male	Cognitive assessment tool	Methods of assessment of Fluoride exposure	Water fluoride level at baseline (mg/L)	Results (Outcome and significance)	Study Quality (score and classification)
			100%			0.25	IQ Level = 104.25±20.73<br><br>Significance: $p < 0.0004$<br>Negative Association	
**Kumar et al. [47]**	2021<br>India	Cross-sectional<br><br>School	480<br><br>10 - 12<br><br>50%	Ravens Standardized Progressive Matrices Test	Drinking water	Low - 1.5<br><br>Medium - 3<br><br>High >5	Mean IQ =15.26±3.40;<br><br>Mean IQ = 12.91±8.14;<br><br>Mean IQ = 9.18±6.55<br><br>Significance: $p < 0.001$<br>Negative Association	68.8<br><br>Fair
**Kundu et al. [48]**	2015<br>India	Cross-sectional<br><br>School	200<br><br>10.33<br><br>50%	Ravens Standardized Progressive Matrices Test	Drinking water	NR	Mean IQ = 85.80±18.854; 76.20±19.101<br><br>Significance: $p < 0.001$<br>Negative Association	66.2<br><br>Fair
**Li, J et al. [28]**	2008<br>China	Cross-sectional<br><br>Hospital	91<br><br>39.3 weeks<br><br>50.5%	Standard neonatal behavioural neurological assessment (NBNA)	Drinking water	1.7 - 6.0<br><br>0.5–1.0	Total NBNA Score 36.48±1.09<br><br>Control 38.28±1.10<br><br>Significance: $p < 0.05$<br>Negative Association	35.1<br><br>Poor
**Li, Y et al. [65]**	2008<br>Mongolia	Cross-sectional<br><br>School	956<br><br>6 - 13	Chinese Standardized Raven Test	Drinking water	NR	Average IQ Endemic - 92.07±17.12;	31.2<br><br>Poor

Study Reference	Year, Country	Study design and setting	Initial sample size, age (year) and % male	Cognitive assessment tool	Methods of assessment of Fluoride exposure	Water fluoride level at baseline (mg/L)	Results (Outcome and significance)	Study Quality (score and classification)
			50.6%				Control -93.78±14.30<br><br>Significance: $p < 0.05$<br>Negative Association	
**Lou et al. [29]**	2020<br>China	Cross-sectional<br><br>School	99<br><br>8 – 12<br><br>50%	Wechsler Intelligence Scale for Children Revised in China (WISC-CR)	Coal burning	NR	Total IQ - 88.51 ± 12.77; 96.64 ± 11.70<br><br>Significance: $p < 0.05$<br>Negative Association	58.4<br><br>Fair
**Lu et al. [30]**	2000<br>China	Cross-sectional<br><br>Community	118<br><br>10 - 12<br><br>NR	Chinese Combined Raven's Test, Copyright 2 (CTR-C2)	Drinking water	0.37±0.04<br><br>3.15±0.61	Mean IQ (±SD)<br>103.05 ± 13.86<br><br>92.27 ± 20.45<br><br>Significance: $p < 0.005$<br>Negative Association	28.6<br><br>Poor
**Martínez et al. [57]**	2016<br>Mexico	Cross-sectional<br><br>Community	132<br><br>NR<br><br>NR	Wechsler Intelligence Scale for Mexican Children (WISC-RM)	Drinking water	NR	Significance:<br>Negative Association	18.2<br><br>Poor
**Qin et al. [31]**	2008<br>China	Cross-sectional<br><br>Community	447<br><br>9 – 10.5<br><br>51%	Raven's Standard Progressive Matrices	Drinking water	0.5 - 1<br><br>0.1 - 0.2	Intelligence Rank in %<br>Rank 1 1.26<br>Rank 2 5.66<br>Rank 3 50.94<br>Rank 4 28.93<br>Rank 5 13.21<br><br>Rank 1 0<br>Rank 2 4.08<br>Rank 3 23.13<br>Rank 4 45.58<br>Rank 5 27.21	24.7<br><br>Poor

Study Reference	Year, Country	Study design and setting	Initial sample size, age (year) and % male	Cognitive assessment tool	Methods of assessment of Fluoride exposure	Water fluoride level at baseline (mg/L)	Results (Outcome and significance)	Study Quality (score and classification)
						2.1 - 4	Rank 1 0.71<br>Rank 2 2.13<br>Rank 3 21.28<br>Rank 4 37.59<br>Rank 5 38.30]<br><br>Significance: p >0.05<br>Negative Association between high and normal and normal and low.	
**Razdan et al. [49]**	2017<br>India	Cross-sectional<br><br>Community	219<br><br>12 - 14<br><br>50%	SPM Test by Raven (1998).	Drinking water	0.60<br><br>1.70<br><br>4.99	Mean IQ Score<br><br>38.60±6.33<br><br>18.94±4.38<br><br>13.94±5.13<br><br>Significance: p < 0.001<br>Negative Association	51.9<br><br>Fair
**Ren et al. [32]**	2008<br>China	Cross-sectional<br><br>School	160<br><br>8 - 14<br><br>51.8%	Wechsler Intelligence Test	Drinking water	NR	Mean IQ = 64.8 ± 20.4<br><br>Significance: p < 0.01<br>Negative Association	13<br><br>Poor
**Rocha-Amador et al. [58]**	2007<br>Mexico	Cross-sectional<br><br>Community	132<br><br>8.1<br><br>50.6%	Wechsler Intelligence Scale for intelligence in children - Revised Mexican Version (WISC-RM)	Drinking water	0.8±1.4<br><br>5.3±0.9<br><br>9.4±0.9	Full IQ Co-efficient 10.2<br><br>11.2<br><br>6.7	44.2<br><br>Poor

Study Reference	Year, Country	Study design and setting	Initial sample size, age (year) and % male	Cognitive assessment tool	Methods of assessment of Fluoride exposure	Water fluoride level at baseline (mg/L)	Results (Outcome and significance)	Study Quality (score and classification)
							Significance: $p < 0.001$<br>Negative Association	
**Saeed et al. [67]**	2020<br>Pakistan	Cross-sectional<br><br>Community	148<br><br>5 - 16<br><br>72.5%	Wechsler Intelligence Scale for intelligence in children	Drinking water	5.64±3.52<br><br>0.15±0.13	IQ Score<br>97.26±15.39<br><br>100.93 ±13.1<br><br>Significance: $p < 0.2$<br>Negative Association	59.7<br><br>Fair
**Saxena et al. [50]**	2012<br>India	Cross-sectional<br><br>School	162<br><br>12<br><br>51.6%	Raven's Standard Progressive Matrices	Drinking water	<1.5<br>1.5 - 3<br><br>3.1 - 4.5<br><br>> 4.5	Mean Intelligence Grade<br>3.16<br>3.85<br><br>4.23<br><br>4.45<br><br>Significance: $p < 0.01$<br>Negative Association	49.4<br><br>Poor
**Sebastian et al. [51]**	2015<br>India	Cross-sectional<br><br>Community	405<br><br>10 - 12<br><br>NR	Raven's Color Progressive Matrices	Drinking water	0.40<br><br>1.2<br><br>2.0	Mean IQ<br>86.37<br><br>88.6<br><br>80.49<br><br>Significance: $p < 0.01$<br>Negative Association	57.1<br><br>Fair
**Seraj et al [64]**	2012<br>Iran	Cross-sectional<br><br>Community	293<br><br>6 - 11<br><br>45.2%	Raven's Color Progressive Matrices	Drinking water	0.8±0.3<br><br>3.1±0.9	Mean IQ<br>97.77<br><br>89.03	49.4<br><br>Poor

Study Reference	Year, Country	Study design and setting	Initial sample size, age (year) and % male	Cognitive assessment tool	Methods of assessment of Fluoride exposure	Water fluoride level at baseline (mg/L)	Results (Outcome and significance)	Study Quality (score and classification)
						5.2±1.1	88.58<br><br>Significance: $p = 0.001$<br>Negative Association	
**Sharma et al. [52]**	2016<br>India	Cross-sectional<br><br>School	270<br><br>10 - 14<br><br>52.2%	Raven's standard progressive matrices	Drinking water	0.40 - 0.68	Intelligence Assessment Scores<br>Grade 1 – 0%<br>Grade 2 – 21%<br>Grade 3 – 83.3%<br>Grade 4 – 8.8%<br>Grade 5 – 0%<br><br>Significance: $p > 0.05$<br>Negative Association	46.8<br><br>Poor
**Soto et al. [59]**	2019<br>Mexico	Cross-sectional<br><br>Community	161<br><br>9 - 10<br><br>54.6%	Raven's Colored Progressive Matrices	Drinking water	0.79 - 1.48	Intellectual Grades; No of Children<br><br>Grade I - 6<br>Grade II - 44<br>Grade III - 79<br>Grade IV - 28<br>Grade V – 4<br><br>Significance: $p = 0.6$<br>Positive Association	71.4<br><br>Good
**Till et al. [62]**	2020<br>Canada	Cross-sectional<br><br>Community	151<br><br>3.51<br><br>47.5%	Wechsler Primary and Preschool Scale of Intelligence-III	Drinking Water, Breast milk & formula feed	0.58±0.08<br><br>0.59±0.07	Full-Scale IQ<br>109.9±12.4<br><br>106.1±15.8<br><br>Significance: $p <0.05$<br>Negative Association	83.1<br><br>Good

Study Reference	Year, Country	Study design and setting	Initial sample size, age (year) and % male	Cognitive assessment tool	Methods of assessment of Fluoride exposure	Water fluoride level at baseline (mg/L)	Results (Outcome and significance)	Study Quality (score and classification)
**Trivedi et al. [53]**	2007<br>India	Cross-sectional<br>School	190<br>12 - 13<br>62.1%	Custom-designed questionnaire	Drinking Water	2.01±0.009<br>5.55±0.41	IQ Score Mean<br>104.44<br>91.72<br>Significance: $p < 0.001$<br>Negative Association	46.8<br>Poor
**Wang et al. [33]**	2020<br>China	Cross-sectional<br>School	325<br>10.05<br>42.46%	Chinese version of the Conners' Parent Rating Scale-Revised (CPRS-48)	Drinking water, urinary Fluoride	NR	Mean IQ scores<br>123.81 ± 12.52<br>Significance: $p < 0.001$<br>Negative Association	64.9<br>Fair
**Wang G et al. [34]**	2008<br>China	Cross-sectional<br>Community	230<br>4 – 7<br>50.8%	Wechsler Preschool and Primary Scale of Intelligence (WPPSI) guidelines	Drinking Water	1.00 - 8.60<br>0.58-1.00	Mean IQ<br>95.64±14.34<br>101.23±15.84<br>Significance: $p < 0.05$<br>Negative Association	41.6<br>Poor
**Wang M et al. [35]**	2019<br>China	Cross-sectional<br>Community	571<br>9.8<br>51.1%	Combined Raven's Test- The rural China	Drinking Water	1.39 ± 1.01	Mean IQ<br>106.74 ± 11.82<br>Significance: $p < 0.01$<br>Negative Association	71.4<br>Good
**Wang S et al. [36]**	2008<br>China	Cross-sectional<br>Community	226<br>7 - 12	Chinese version of the Raven's standard theoretical intelligence test	Coal burning	NR	Distribution of Intelligence Rankings<br><5% = 7<br>5 – 24% = 12	29.9<br>Poor

Study Reference	Year, Country	Study design and setting	Initial sample size, age (year) and % male	Cognitive assessment tool	Methods of assessment of Fluoride exposure	Water fluoride level at baseline (mg/L)	Results (Outcome and significance)	Study Quality (score and classification)
			56.5%				25 – 74% = 36<br>≥75% = 2<br><br><5% = 6<br>5 – 24% = 24<br>25 – 74% = 59<br>≥75% = 8<br><br><5% = 2<br>5 – 24% = 4<br>25 – 74% = 29<br>≥75% = 14<br><br>Significance: $p < 0.01$<br>Negative Association	
**Wang SX et al. [37]**	2007<br>China	Cross-sectional<br>Community	376<br>9.9<br>50.9%	Combined Raven's Test- The rural China	Drinking water	8.3 ± 1.9	Mean IQ<br>100.5 ± 15.8<br><br>Significance: $p < 0.05$<br>Negative Association	68.8<br>Fair
**Wei et al. [38]**	China	Cross-sectional<br>School	741<br>8 – 12<br>NR	Raven's Standard Progressive Matrices - revised for China	Coal burning	NR	Intellectual Deficiency = 8.4%<br>Below Average = 32.2%<br>Average =39.6%<br>Good = 19.1%<br>Superior = 0.7%<br><br>Intellectual Deficiency = 9.7%<br>Below Average = 34.8%<br>Average =41.0%<br>Good = 14.5%<br>Superior = 0.0%	32.5<br>Poor

Study Reference	Year, Country	Study design and setting	Initial sample size, age (year) and % male	Cognitive assessment tool	Methods of assessment of Fluoride exposure	Water fluoride level at baseline (mg/L)	Results (Outcome and significance)	Study Quality (score and classification)
							Intellectual Deficiency = 0<br>Below Average =2.9%<br>Average =33.7%<br>Good =60.6%<br>Superior =2.9%<br><br>Significance: p >0.05<br>Positive Association	
**Xiang et al. [39]**	2003<br>China	Cross-sectional<br><br>Community	734<br><br>11.1<br><br>55%	Combined Raven's Test for Rural China	Drinking Water	2.47 ± 0.79<br><br><1.0<br><br>1.0–1.9<br><br>2.0–2.9<br><br>3.0–3.9<br><br>>3.9<br><br>0.36 ± 0.15	Mean IQ ± SD<br><br>92.02±13.00<br><br>99.56±14.13<br><br>95.21±12.22<br><br>92.19±12.98<br><br>89.88±11.98<br><br>78.38±12.68<br><br>100.41±13.21<br><br>Significance: p <0.01<br>Negative Association	54.5<br><br>Fair
**Xu et al. [40]**	2020<br>China	Cross-sectional<br><br>School	633<br><br>10.1<br><br>46.4%	Combined Raven's Test-The Rural in China method	Drinking Water	< 1.0 (control)<br><br>> 1.0 (only prenatal excessive)	Mean IQ Score<br>123.92 ± 12.50<br><br>119.76 ± 11.28	87.0<br><br>Excellent

Study Reference	Year, Country	Study design and setting	Initial sample size, age (year) and % male	Cognitive assessment tool	Methods of assessment of Fluoride exposure	Water fluoride level at baseline (mg/L)	Results (Outcome and significance)	Study Quality (score and classification)
						> 1.0 (only childhood excessive)<br><br>> 1.0 (both prenatal and childhood excessive)	124.65 ± 10.88<br><br>123.04 ± 11.24<br><br>Significance: Positive Association	
**Yang et al. [41]**	2008<br>China	Cross-sectional<br><br>Community	60<br><br>8 - 14<br><br>NR	Chinese Comparative Scale of Intelligence Test (Third Edition)	Drinking Water	2.97<br><br>0.5	Average IQ scores<br><br>76.67±7.75<br><br>81.67±11.97<br><br>Significance: p >0.05 Positive Association	39<br><br>Poor
**Yu et al. [42]**	2018<br>China	Cross-sectional<br><br>Community	2886<br><br>9.9<br><br>52.6%	Combined Raven's Test – The Rural in China method (CRT-RC2)	Drinking Water	2.00 ± 0.75<br><br>0.50 ± 0.27	Mean IQ<br>106.4 ± 12.3<br><br>107.4 ± 13.0<br><br>Significance: p = 0.03 Negative Association	66.2<br><br>Fair
**Zhao et al. [43]**	2021<br>China	Cross-sectional<br><br>School	567<br><br>9.15<br><br>50.1%	Combined Raven's Test (modified in China)	Drinking Water	1.53–2.84	Mean IQ scores<br><br>112.17 ±11.75<br><br>Significance: Negative Association	79.2<br><br>Good

Study Reference	Year, Country	Study design and setting	Initial sample size, age (year) and % male	Cognitive assessment tool	Methods of assessment of Fluoride exposure	Water fluoride level at baseline (mg/L)	Results (Outcome and significance)	Study Quality (score and classification)
**Zhao LB et al. [44]**	1996<br>China	Cross-sectional<br><br>Community	320<br><br>7 - 14<br><br>50%	Official intelligence quotient (IQ) tests	Drinking Water	4.12<br><br>0.91	Mean IQ scores<br><br>97.69±13.00<br><br>105.21±14.99<br><br>Significance: p <0.01<br>Negative Association	20.8<br><br>Poor
